# Benchmarking Neural Network Personalized Musculoskeletal Hand Models Against Current Personalization Standards Using Experimental Magnetic Resonance Imaging and Fine-Wire Electromyography

**DOI:** 10.1109/TNSRE.2026.3711799

**Published:** 2026

**Authors:** Maximillian T. Diaz, Erica M. Lindbeck, Alexis R. Benoit, Isaly Tappan, Joel B. Harley, Jennifer A. Nichols

**Affiliations:** J. Crayton Pruitt Family Department of Biomedical Engineering, University of Florida, Gainesville, FL 32611 USA; Department of Electrical and Computer Engineering, University of Florida, Gainesville, FL 32611 USA; J. Crayton Pruitt Family Department of Biomedical Engineering, University of Florida, Gainesville, FL 32611 USA; Department of Electrical and Computer Engineering, University of Florida, Gainesville, FL 32611 USA; Department of Electrical and Computer Engineering, University of Florida, Gainesville, FL 32611 USA; J. Crayton Pruitt Family Department of Biomedical Engineering, University of Florida, Gainesville, FL 32611 USA

**Keywords:** artificial intelligence, biomechanics kinematics, kinetics, motion capture, muscle activity, musculoskeletal system

## Abstract

Physics-based musculoskeletal models are often used to understand complex systems, like the human hand. Because generic hand models typically represent less than 1% of the population, models are often personalized to match individual people. Multiple personalization approaches exist. Yet, it is unknown which methods are best for studying hand tasks. Here we i) apply five musculoskeletal personalization methods to hand models and ii) evaluate the methods by comparing simulated muscle activations to measured electromyography. The five personalization methods [scaling, joint moment optimization using Neuromusculoskeletal Modeling Pipeline (NMSM), magnetic resonance imaging (MRI) segmentation, MRI segmentation combined with joint moment optimization, and a novel neural network] were used to create 14 personalized models for each participant (n = 13 healthy adults). The models were then used to simulate seven range of motion and eight isometric hand tasks in the inverse direction using static optimization. In contrast to current personalization methods that require extensive experimental data and processing, we demonstrated musculoskeletal hand models could be personalized using only lateral pinch force data and a custom neural network. None of the personalization methods resulted in anatomically accurate muscle parameter sets, when compared to the anatomical standard of MRI segmentation. However, the neural network method led to significantly lower errors when predicting muscle activations compared to current state-of-the-art methods for personalizing musculoskeletal model parameters without personalizing associated neural control models. By reducing the experimental data and time barriers required to create personalized hand models, neural networks may allow for wider use of musculoskeletal models in clinical settings.

## I. Introduction

Musculoskeletal models can enable detailed investigation into the biomechanics of complex structures, such as the hand. This detailed investigation can estimate joint forces, muscle forces, and anatomical changes that cannot be directly measured [1], [2], provide the ability to augment participant populations [3], [4], [5], [6], or enable generation of synthetic datasets [3], [7], [8]. Musculoskeletal models leverage physics-based modeling using Newtonian mechanics, with bones defined as rigid bodies and muscles defined as force actuators using the Hill-type muscle model. Generic musculoskeletal models often represent a 50th percentile male with measured muscle parameters derived from a mixture of cadaveric and *in vivo* experiments [9], [10], [11], [12]. Unfortunately, less than 0.5% of the population is represented by a 50th percentile male model [13]. To better represent individuals, generic models can be personalized to individuals. The simplest personalization methods linearly scale a generic model’s length and mass parameters to match those of an individual [2], while more advanced methods use optimization to improve prediction accuracy and/or medical imaging to improve the anatomical accuracy of models.

Optimization approaches for personalizing musculoskeletal models focus on tuning model parameters with experimental data to increase prediction accuracy. However, the optimization criteria and data can vary widely between methods. For example, Modenese et al. [14] used model posture to tune length-based parameters of scaled OpenSim models. Specifically, they optimized the musculotendon length to ensure that the operating range of the muscles were preserved following scaling. In contrast, the Neuromusculoskeletal Modeling Pipeline (NMSM) adjusts model parameters by reducing the error between predicted joint moments from forward simulations and predicted joint moments from inverse simulations [15], [16]. While each optimization method uses a different type of input data, each approach allows for improving prediction accuracy by selecting anatomically inaccurate parameter sets. These optimization approaches have been shown to provide realistic predictions. However, model performance is often evaluated without direct comparison to an experimental ground truth. For example, common evaluation methods involve i) using joint forces as an indirect assessment of muscle parameter estimates [15], [17], [18], ii) using limited tasks, which can result in similar tasks being used in both model tuning and testing [15], [19], or iii) by using a generic model as the baseline comparator when experimental data is not available [20].

In contrast, magnetic resonance imaging (MRI) is widely considered to be the most anatomically accurate personalization method since individual musculoskeletal structures can be obtained via segmentation. The extent of MRI personalization varies since a combination of muscle parameters [21], [22], bone morphologies [23], and tendon insertions [21], [22] can be adjusted. For example, Scheys et al. [23] used MRI to personalize leg bone geometry producing significantly different inverse kinematics results compared to an anthropometrically scaled model. Hainisch et al. [22] and Charles et al. [21] both used MRI to compute personalized muscle parameters and wrapping paths to personalize models in children and adults, respectively. Hainisch et al. [22] found MRI personalization leads to more accurate joint moments compared to scaled models, and Charles et al. [21] found MRI personalized models predicted isokinetic ankle, knee, and hip torques more accurately than scaled models. However, even though it is considered a robust method, MRI personalization cannot directly measure the key muscle parameter of tendon slack length, and this method relies on assumed values for specific tension and optimal sarcomere length [21], [24]. Unfortunately, reported values for optimal sarcomere length and specific tension range from 1.6 to 3.0 *μ*m and 2–137 N/cm^2^, respectively, and vary within an individual [25], [26], [27], [28]. Therefore, to adjust for uncertainty in chosen values, MRI personalization is often combined with optimization methods (e.g., MRI+NMSM) to improve parameter estimates [20].

While personalization methods have been widely developed for the lower limb, the human hand has unique structures and functions not seen elsewhere in the body that have limited work to develop personalized hand models. For humans to simply interact with the environment, they must control 30 muscles, to move 27 bones, to perform one of over 33 possible grips [29], [30], [31]. It is such a high level of complexity that the human brain had to further evolve before unlocking the hand’s full potential [29]. A key to the hand’s functional success is found in its muscle structure, which includes intrinsic muscle bellies within the hand and extrinsic muscle bellies within the forearm [29], [30], [32], [33]. Because both intrinsic and extrinsic muscles are arranged in tight, compact layers, fine-wire electromyography (EMG) is often required to experimentally measure hand muscle activity [33]. Furthermore, the hand has a high degree of muscle redundancy, including multiarticular muscles that control multiple joints and many muscle acting across each joint. This results in a semi-infinite number of ways to control the hand in combination with a limited surface area for placing fine-wire EMG or other sensors. Unfortunately, the current limitations of experimental hand data make it clinically difficult to fully personalize neurological model parameters, and musculoskeletal parameters remain time-consuming to directly measure [34], [35], [36], [37], [38].

Given the complexity of the hand, few studies have implemented personalization approaches. For example, Han et al. [38] addressed the issue of complexity by limiting their joint angle optimized hand models to single-plane motions. In contrast, Goislard de Monsabert et al. [39] limited their study to isometric flexion and extension tasks across the fingers and wrist when personalizing a global specific tension and muscle compartment scaling factors. Of note, Goislard de Monsabert et al. [39] also optimized the neural control, but had to do a task-specific optimization and used the same data for tuning and validation. To overcome the challenges of obtaining and processing experimental hand data, we previously developed a neural network that personalizes musculoskeletal models of the upper limb using data from a single lateral pinch in combination with height and weight [3]. As previously described, the neural network was developed with a synthetic population (n=50,000) simulating lateral pinch [3]. Briefly, the neural network was trained using height, weight, and the lateral pinch force’s x-, y, and z-components as inputs to predict a percentage change (as measured from the generic model) for five parameters: rigid body mass, pennation angle, optimal fiber length, maximum isometric force, and tendon slack length. A personalized model was then created by applying the predicted percentage change to all segments or muscle for each given parameter. As detailed in Lindbeck et al. [3], this approach lead to a personalization that improves prediction accuracy without enforcing strict anatomical accuracy. The original development and analysis were limited to synthetic populations with simulated data, so it is unknown to what extent this method can be applied to experimental data.

Therefore, in this study, we i) compare the muscle parameter sets created from different musculoskeletal model personalization methods at the hand and ii) use experimentally measured fine-wire EMG to determine which models provide the most accurate predictions for inverse simulations to predict muscle activation. We specifically compare generic, scaling, optimization (NMSM), MRI, MRI with optimization, and neural network personalization approaches. We also acknowledge that this study evaluates the effects of personalizing musculoskeletal modeling parameters without additional steps toward personalizing the neural control model.

## II. Methods

**Fig. 1.**
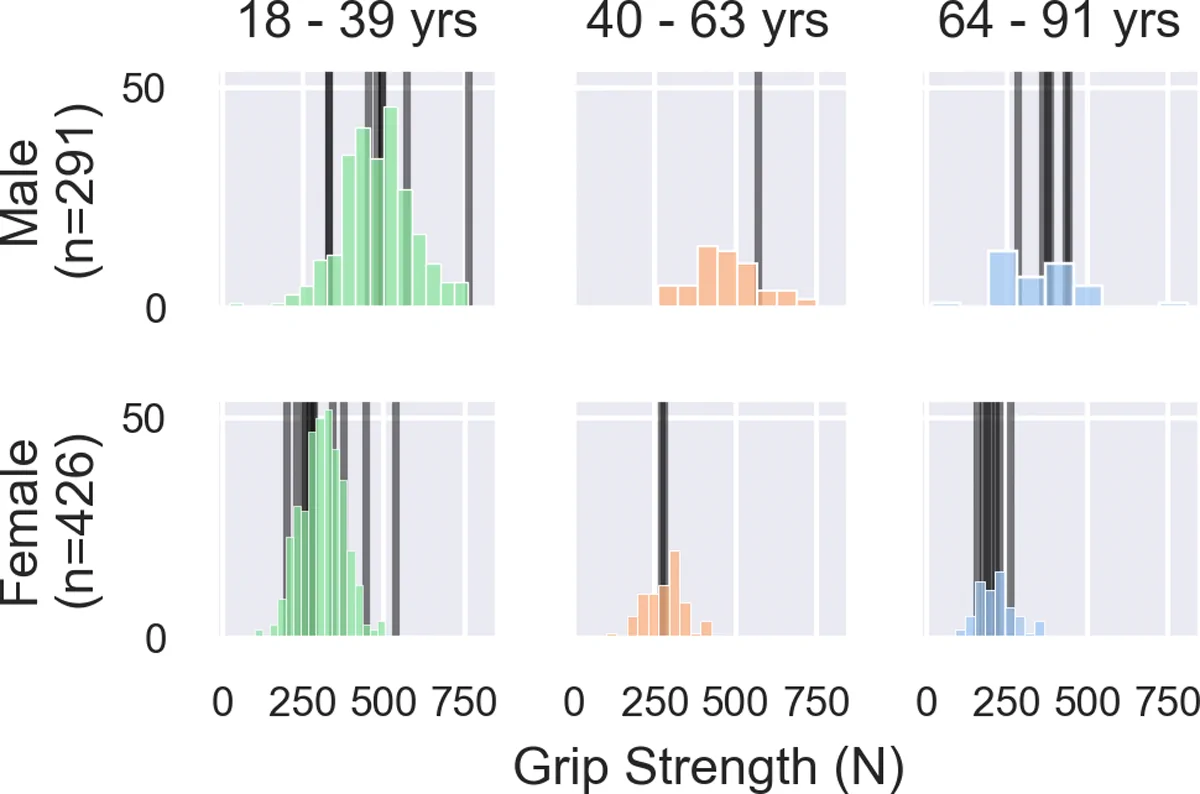
Grip strength sub-samples (colored) used to determine recruited individuals (black) [40], [41]. Biomechanics participants were recruited by originally trying to target an individual from the mean, ±1, and ±2 standard deviations for each sub-group. However, due to low recruitment rates from participants between 40–63 years, and male participants over 64 years, the recruitment criteria changed focus to capture the population of individuals under 39 years and over 64 years.

Thirty participants [n=30; age 48.6 ± 24.9 years; 14 male, 16 female; 26 right hand dominant, 4 left hand dominant] were recruited to take part in an IRB-approved study (UF IRB #202002061) to measure muscle activations, joint angles, and external forces during hand tasks. Individuals were selectively recruited to obtain equal representation from previously reported grip strength distributions for young, middle-aged, and older adults (Fig. 1) [40]. The experimental data, generic model, and scaled models described below are publicly available on Kaggle [40], [41]. Note, throughout this paper the word model only refers to musculoskeletal models, parameters only refer to values used to define variables within musculoskeletal models, and networks refers only to neural networks. The full experimental protocol is described in Diaz et al. [40], so only the tasks and data used within this study are described herein.

### A. Experimental Data Acquisition and Processing

EMG and motion capture were used to record muscle activity and movement. Fine-wire electrodes recorded data from 8 thumb muscles (Delsys; 3,000 Hz). Electrodes were placed under ultrasound guidance (SuperSonic Imagine Mach 30) in four extrinsic [*flexor pollicis longus* (FPL), *extensor pollicis longu*s (EPL), *extensor pollicis brevis* (EPB), and *abductor pollicis longus* (APL)] and four intrinsic [*flexor pollicis brevis* (FPB), *adductor pollicis* (ADD), *opponens pollicis* (OPP), and *first dorsal interossei* (FDI)] muscles [33]. Motion capture was recorded with a 12-camera system (Vicon Vero; 100 Hz) and a custom marker set (Fig. 3). Immediately after sensor placement, muscle-specific maximum voluntary contractions (MVCs) were performed to facilitate normalizing recorded EMG data [42]. Each MVC was performed 5 times for 5 seconds with 5 second rests between each exertion; a trained experimentalist provided resistance during each MVC using standard procedures [32], [33]. A static calibration pose was also recorded; during this pose, participants were seated with their arm extended in front of them without support [shoulder flexion: 90°, elbow flexion: 0°, forearm supination: 0°, wrist deviation: 0°, hand relaxed with fingers straight and spread].

**Fig. 2.**
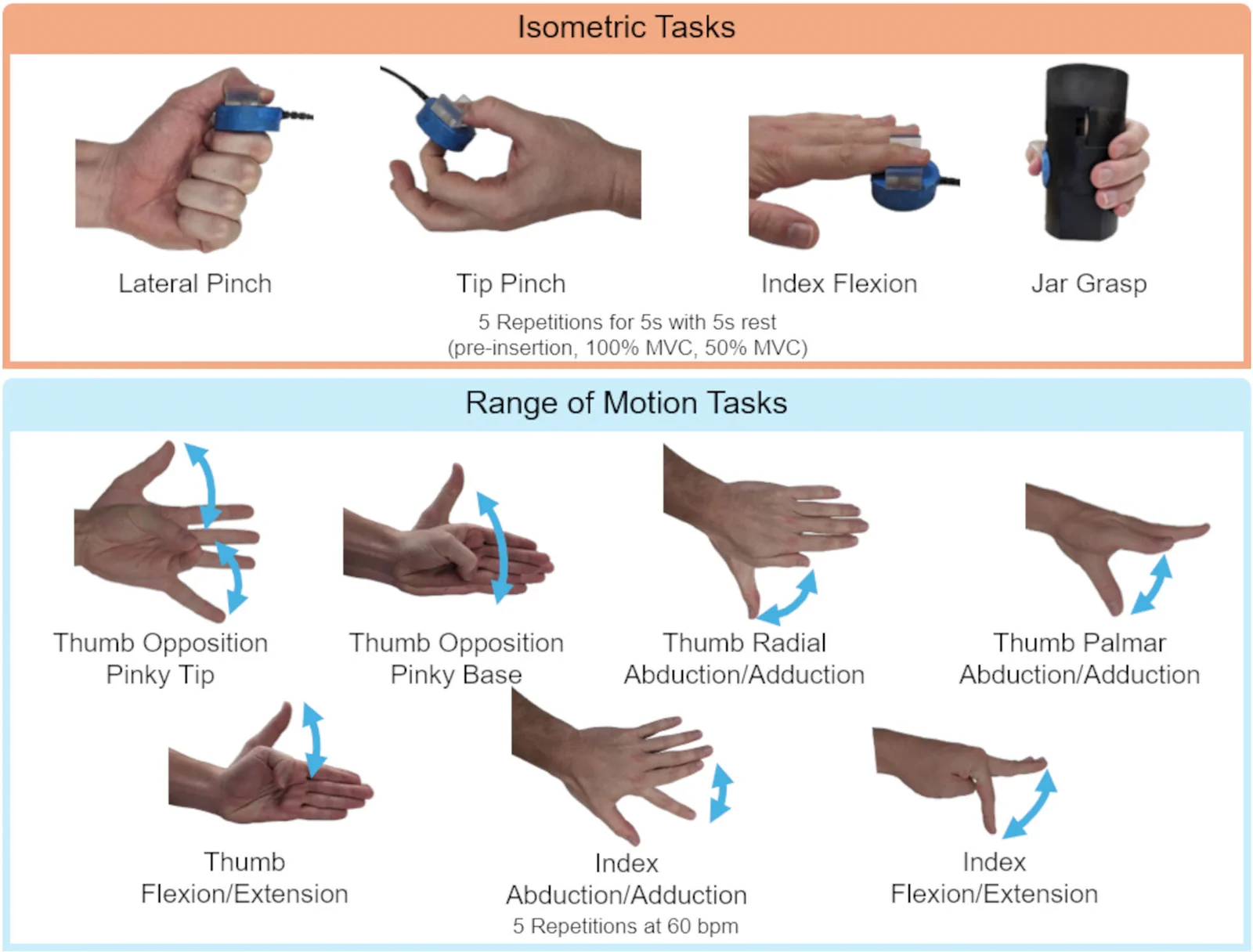
Common isometric and range of motion tasks performed by the human hand. Two repetitions of each task were selected for personalizing musculoskeletal model with the remaining three used to evaluate model performance when predicting muscle activations.

**Fig. 3.**
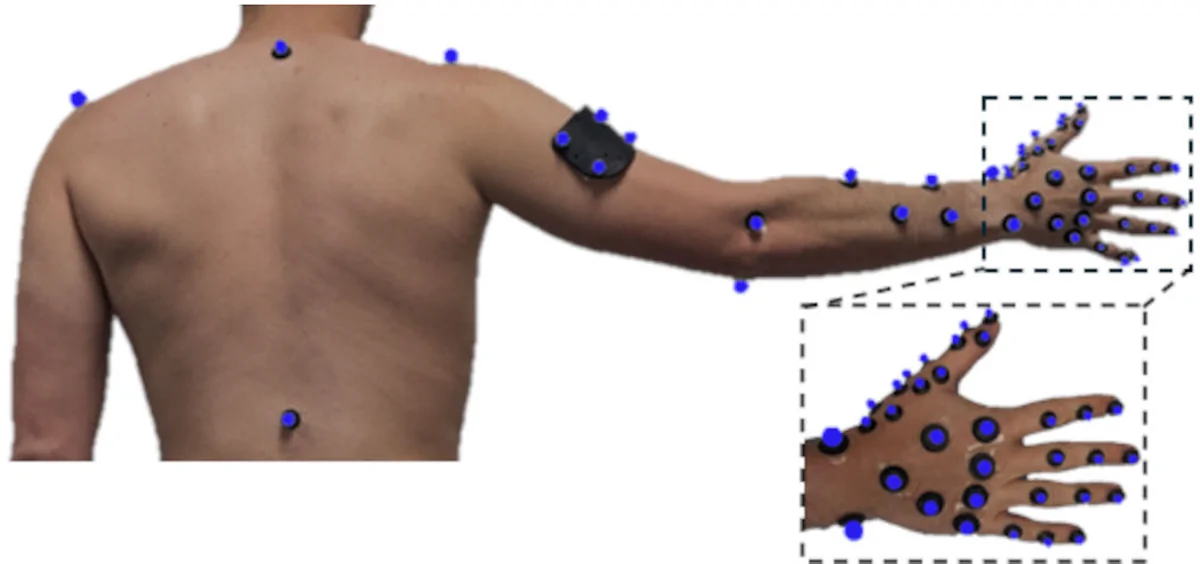
Posterior view of motion capture marker set (sternum marker not shown) used to track the thorax, arm, forearm, hand, thumb, and digits. The thumb was tracked with six markers on the first metacarpal [three proximal head, three distal head], three markers on the proximal phalanx, and three markers on the distal phalanx. The fingers were tracked with a single marker on each of the distal metacarpal head, proximal, medial, and distal phalanx. Tracking clusters were placed on the hand, forearm, upper arm, and thorax [acromion, sternal notch, C7, T12]. Additional scaling makers were placed on the radial styloid, ulnar styloid, medial elbow, and lateral elbow. The forearm cluster varied between subjects to accommodate wires and boxes (not shown) required for fine wire EMG.

Following EMG insertions and motion capture calibration, each participant performed isometric and range of motion tasks to capture hand and thumb functions. Each range of motion task (Fig. 2) was performed 5 times to a metronome beat (60 bpm) with the arm in the same position as the static posture. Each isometric task (Fig. 2) was then performed at 100% and 50% MVC for 5 seconds with 5 second rests for 5 repetitions. For the 4 evaluated isometric tasks (lateral pinch, tip pinch, index flexion, and jar grasp), thumb- or finger-tip force was measured with a six-degree-of-freedom, time-series force sensor (ATI Mini40; 3,000Hz) [43]. The center of the force sensor was aligned with the center of the finger pad such that the sensor’s transducer cable aligned with the digit’s anterior-posterior (long) axis. This allows for the location of the force sensor (and measured forces) to be registered to the motion capture data using a virtual marker located in the center of the finger pad.

Force, EMG, and marker data were zero-lag filtered for use in musculoskeletal simulations. The six-degree-of-freedom forces were low-pass filtered (4 Hz, 4th order Butterworth) and rotated 22.5° about the z-axis (dorsal-ventral). The rotation was performed to align the sensor and thumb- or finger-tip reference frames. EMG signals were high-pass filtered (20 Hz, 4th order Butterworth), rectified, low-pass filtered (5 Hz, 4th order Butterworth), and then smoothed with a 0.2 s moving average filter to obtain EMG envelopes [8], [44], [45]. EMG signals were then normalized using the maximum value recorded across all isometric tasks and muscle-specific MVC tasks. Gap-filled marker trajectories were low-pass filtered (6 Hz, 4th order Butterworth) and rotated 180° about the y-axis (superior-inferior) to align the experimental coordinate frame with the OpenSim coordinate frame. Trajectories from left-handed participants were mirrored about the sagittal plane to simulate a right arm. Joint angles were calculated with inverse kinematics performed in OpenSim 4.4 [46]. Trial start-stop times were determined using a Gaussian mixture model to perform binary classification on the recorded forces for isometric tasks and joint angles for range of motion tasks. Gaussian mixture models were used opposed to traditional thresholding as Gaussian mixture models have been found to be better for batch processing large datasets [47], [48].

### B. MRI Acquisition and Processing

A structural MRI of the arm and forearm were taken two weeks after the biomechanics session from a subset of 15 participants [n = 15; age: 46.1 ± 23.3 years; 7 male, 8 female; 12 right hand dominant, 3 left hand dominant] (UF IRB #202202865). Eligibility was determined by 1) MRI exclusion criteria (e.g., ferrous implants, claustrophobia, etc.) [36], 2) quality of experimental biomechanics data, and 3) maintaining demographic representation. Participants were placed supine in the scanner while 3T axial MRIs were acquired (ST: 3mm, TR: 3,440 ms, TE: 20 ms, FA: 90°, FOV: 11cm, matrix: 224 × 224, bandwidth: ±1 kHz) (3 T/70 cm Philips MR7700) [49], [50]. Forearm muscles and bones were segmented in 3D Slicer [51].

Optimal fiber length and maximum isometric force were calculated for 27 of the 69 muscles included in the MoBL-ARMS upper limb and ARMS hand and wrist models (Supplemental Table I) [9], [10], [11], [12]. Muscle length and volume were computed from MRI muscle segmentations using 3D Slicer’s Analysis Tool [51]. Optimal fiber length and maximum isometric force were then calculated according to Equations (1) to (3) with fiber length to muscle length ratios taken from Holzbaur et al. [25] and specific tension assumed to be 50.8 N/cm^2^ [12]. For muscles that contained multiple tendons [FDS, FDP, EDC, ECR], the parameters of the individual model actuators (e.g., FDSI, FDSM, FDSR, FDSP) were calculated using the ratios for optimal fiber length and maximum isometric force previously employed to obtain the sub-components of each larger muscle [25], [49].

(1) $$l_{o}^{M}\;=l_{\mathrm{meas}\;}^{M}{\left(\frac{l^{M}}{l_{o}^{\dot{M}}}\right)}^{-1}$$

(2) $$PCSA\;=\frac{V_{\mathrm{meas}\;}^{M}}{l_{o}^{M}}$$

(3) $$F_{o}^{M}\;=PCSA*\sigma$$

### C. Model Development and Personalization

**Fig. 4.**
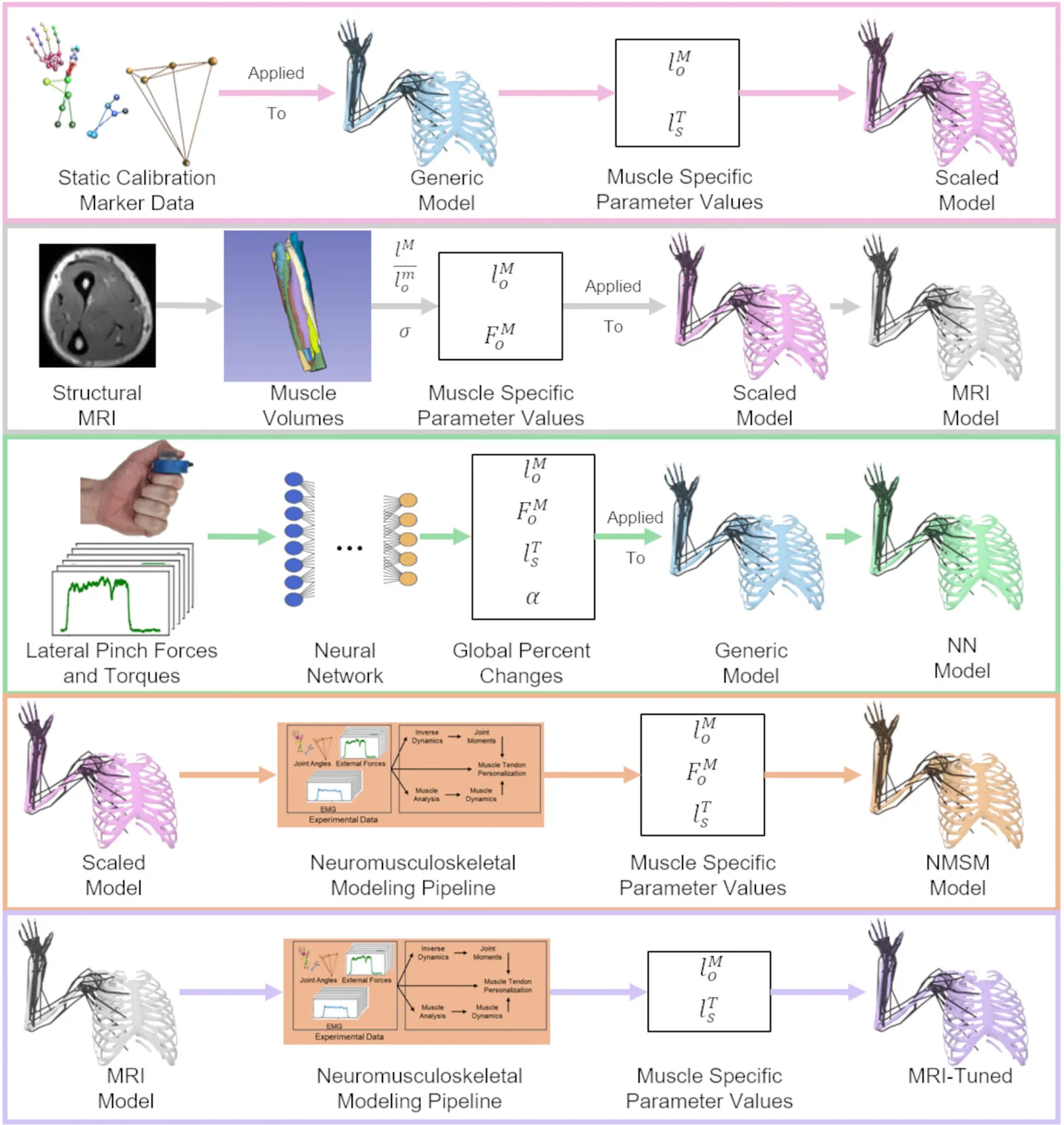
Development pipeline for five personalization methods to create the final 14 personalized hand models for each participant.

Data from 13 participants [n = 13; age 43.0 ± 23.4 years; 7 male, 6 female; 12 right hand dominant, 1 left hand dominant] were used to evaluate personalization methods. The processed experimental data (joint angles, EMG, external forces, and MRIs) were used to create 14 personalized musculoskeletal models for each participant in OpenSim 4.4 (Fig. 4). The generic model and scaled model were the same models derived from previously published upper limb models used to process the motion capture data. As described in detail below, five musculoskeletal models were created using the NMSM Pipeline by varying the tuning data. Five musculoskeletal models were made with the neural network (NN) using each post-insertion lateral pinch 100% MVC trial. An MRI model was created using values calculated from forearm segmentations. The final model, termed the MRI-tuned model, consisted of tuning the MRI model using NMSM [16], [20]. In all cases, models were created by estimating the muscle parameter sets used within the musculoskeletal models in OpenSim. All OpenSim models used the Millard2012EquilibriumMuscle muscle model [52].

1. *Generic and Scaled Models:* A generic musculoskeletal model was scaled to match participants for inverse kinematics. The generic model was created by combining the MoBL-ARMS upper limb and ARMS hand and wrist models [9], [10], [11], [12] and adding the custom marker set. The generic model was then anthropometrically scaled for each participant using static marker data. All scaling was completed in OpenSim, which computes scale factors as the ratio between experimentally measured marker distances and modeled marker distances [46]. Uniform scale factors based on the long axis of each rigid body (e.g., lateral epicondyle to distal radial head for radius rigid body) were used to scale all three dimensions. The generic and scaled OpenSim models have been previously published [40], [41].
2. *NMSM Models: Five personalized* musculoskeletal models were created using the NMSM parameter optimization method by varying the tuning data. Specifically, NMSM models were trained by optimizing the scaled model’s parameters with i) only 50% MVC, ii) 100% MVC, iii) all isometric tasks, iv) all range of motion tasks, or v) all tasks. Multiple tuning variations were tested since previous works have trained with primarily gait data [15], [16], [20]. However, the hand does not have a baseline task like gait; instead, the hand has over 30 grasps and over 20 degrees-of-freedom representing isometric and isokinetic tasks. Furthermore, given that the hand muscles operate on a very small portion of the forcelength-velocity curve in both isometric and isokinetic tasks, it may be unnecessary to utilize a complex task set to achieve the same level of muscle dynamics calibration seen in lower limb studies. Hence, a researcher may only need a hand model that performs isometric tasks, isokinetic tasks, or both; but if one has an advantage in model tuning then collecting those extra tasks may prove beneficial. Two trials for each task were randomly selected for NMSM tuning, while the remaining three trials of each task were used to evaluate model performance. While the models contained all upper limb muscles, NMSM was set to only include muscles that cross the wrist and/or hand during tuning. This was done as the included tasks did not require any elbow or shoulder muscles, and those joints served to only position the hand in space. Inclusion of muscles across the wrist meant that extrinsic thumb muscles could be tuned, but also that the primary wrist muscles were included. Since the primary wrist muscles did not have EMG data for the specified tasks, NMSM’s default method of principle component analysis was used to accounted for missing EMG data during tuning [16]. To create the five NMSM personalized models, the maximum isometric force, optimal fiber length, and tendon slack length parameters determined by NMSM for each tuning set were used to replace those parameter values in the scaled model for only thumb and index finger muscles. Throughout the paper musculoskeletal models personalized with the parameter sets derived from the NMSM pipeline are referred to as NMSM models.
3. *MRI & MRI-tuned Models: Two personalized models (MRI and MRI-tuned) were created using muscle volumes segmented from forearm MRIs. To create the MRI model, the maximum isometric force and optimal fiber length calculated for the 27 muscle actuators replaced those in the scaled model. The rigid body masses were then adjusted by multiplying the volumes by respective densities for segmented muscles (0.977 g/cm*^3^) [53], segmented fat (0.900 g/cm^3^) [54], and segmented bones (radius, ulna) [average density for age specified in Meema and Meema [55] Table I, 0.937–1.179 g/cm^3^)] [55]. The total mass was divided between the radius and ulna using the original ratios [25]. The calculated radius and ulna mass were then used in the MRI personalized model. Muscle paths were not adjusted given the small and complex behavior of forearm muscles, and previous benchmarking work highlighting a lack of consensus on how to implement personalized paths, wrapping points, and attachment sites [20]. Hand joint centers were also not personalized. The MRI-tuned model was created by replacing the optimal fiber length and tendon slack length with values obtained from running NMSM to optimize the MRI model with all tuning data.
4. *Neural Network Model: A previously developed neural network personalization method was used to adjust the generic* musculoskeletal model’s maximum isometric force, optimal fiber length, tendon slack length, and pennation angle using a participant’s five lateral pinch trials [3]. The participant’s lateral pinch forces, height, and weight were provided as inputs to the neural network, and the network provided a global parameter percent change from the generic musculoskeletal model for each parameter. Each parameter’s global percentage change is applied to all muscles in the generic model to create a personalized musculoskeletal model. For example, if the network estimates a global percentage change of −10% for maximum isometric force, the maximum isometric force for the supinator decreases from 376.9 N to 339.2 N while brachialis decreases from 1177.4 N to 1059.7 N. Since each lateral pinch trial predicts a separate parameter set, five NN personalized musculoskeletal models were created for each participant to account for prediction uncertainty. Throughout the paper, musculoskeletal models personalized with the parameter sets created by the neural network shall be referred to as NN models.

### D. Simulations to Predict Muscle Activations

Personalized musculoskeletal models were evaluated in the inverse direction using static optimization. Static optimization takes the inputs of joint angles and external forces to predict muscle activations. During simulations, the rotation and translations of each personalized model’s thorax were locked allowing only the arm, forearm, and hand to move. Locking the model was performed for computation simplicity, which reduced run times and number of failed simulations. The three testing trials selected when tuning NMSM models were used to evaluate performance for all personalized models. All 8,190 simulations (15 tasks × 3 trials × 14 personalized models × 13 participants) were run in OpenSim 4.4 using a custom Python batch processing code run on HiPerGator, the University of Florida’s high-performance supercomputer (allocation used: 992 CPU cores, 4 GB ram each). The overall computation time for all 8,190 simulations was 4,686 hours. The isometric trials took an average of one hour per trial (4,368 hours total = 1 hour × 13 participants × 14 models × 8 tasks × 3 trials); while range of motion tasks took five minutes per trial (318 hours = 5 minutes × 13 participants × 14 models × 7 tasks × 3 trials / 60 minutes). The predicted muscle activations were zero-lag, low-pass filtered (4 Hz, 4th order Butterworth) to account for computational noise generated by static optimization in OpenSim.

### E. Statistical Analysis

1. *Model Parameters to Assess Anatomic Accuracy: Percent di*ff*erences were calculated between each predicted parameter set* (optimal fiber length, pennation angle, maximum isometric force, and tendon slack length) and the measured MRI parameter set to evaluate the anatomical accuracy of each method. In addition, to determine the influence of NMSM’s tuning data (i.e., tuning with different tasks) on model parameters, percent differences were calculated between the baseline scaled model and each NMSMs personalization type. Within-subject, repeated measures ANOVAs and Bonferroni post-hoc comparisons were performed to determine significance (p<0.05) for each parameter set.
2. *Muscle Activations to Assess Prediction Accuracy: Model performance was evaluated by comparing the predicted muscle activations to the measured EMG*. For this comparison, a phase shift was not introduced to account for electromechanical delay [56]. Root mean squared error (RMSE) was calculated between each prediction and the associated measured data to evaluate overall performance [20]. Since muscle activations are normalized, the values are unitless and range from 0 to 1; therefore, RMSE is also bound from 0 to 1 where a RMSE of 0.1 represents an average error of 10%. Significant differences in RMSE between models were compared with repeated measures ANOVA, followed by a Bonferroni post-hoc comparison. The square of Pearson’s correlation coefficient (r^2^) was also calculated to evaluate differences in curve shape. Note, the square of Pearson’s correlation coefficient measure focuses on the shape of two curves, not the magnitude. Thus, it provides an opportunity to evaluate if predicted muscle activations followed the correct trend, despite differences in magnitude that may have resulted from not optimizing neural controls or potentially not normalizing EMG data to true MVCs.

Differences within individual tasks were evaluated using FPL as a representative muscle, since it is a primary flexor required for isometric thumb tasks and exhibited large changes between model personalization methods. Differences within individual tasks were not evaluated for all muscles as this requires 1,800 comparisons for static optimization; however, visualizations are provided for all RMSE in Supplemental Figures 1 and 2, and calculated r^2^ for the FPL muscle across all models and tasks is in Supplemental Table 2.

The predicted activations from the multiple NN and NMSM model were reduced to a single prediction for NN and NMSM, respectively. The reduction was made to simplify the comparisons from 14 predictions down to six. Reducing the NMSM musculoskeletal models’ predicted activations from five predictions to a single average predicted activation was justified as initial analysis found no significant differences between the five models. Because which of the five models performed best varied between muscle, task, and participant, the five NMSM models were represented by an average NMSM prediction. The average prediction was calculated by taking the average predicted activation from the five NMSM predictions for each time point to calculate the average predicted activation for the task. This averaging was done for each individual muscle and task for each participant. Average NN musculoskeletal model predictions were created for each task in a similar manner. The NN musculoskeletal model predictions were averaged because the NN method produced separate parameter sets based on each of the five experimental trials per participant. Note, this approach minimizes uncertainty in the neural network by averaging the predicted signals [3], [57]. For both musculoskeletal models (NMSM and NN), the average prediction was used to calculate the RMSE and r^2^ of each modeling approach against the experimental EMG data.

## III. Results

### A. Anatomic Accuracy: Comparison of Parameter Sets

**Fig. 5.**
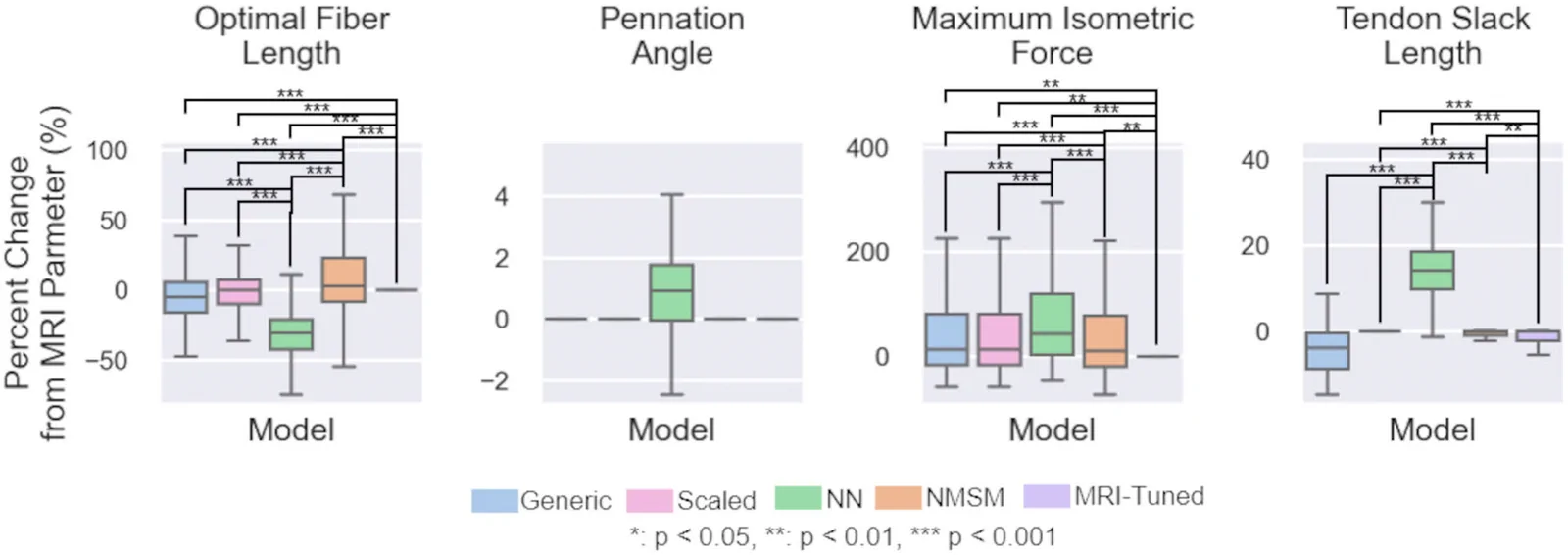
Percent change of personalized muscle parameters compared to the MRI personalized model parameters. The MRI personalized model was selected as the baseline since it is considered the anatomical gold standard. Only the neural network method allowed for the personalization of pennation angle. Additionally, tendon slack length cannot be measured from MRI, so there is no difference between the scaled and MRI values.

Each personalization method resulted in a unique parameter set (Fig. 5). The NN approach resulted in significantly (p<0.05) lower optimal fiber lengths, higher maximum isometric forces, and higher tendon slack lengths compared to the other methods. In contrast, the NMSM approach as well as the generic and scaled models all resulted in similar directional changes relative to MRI parameter values; however, the NMSM parameter changes were significantly (p < 0.05) greater in magnitude than those of the generic or scaled models. Additional tuning of the MRI model with NMSM (MRI-tuned model) primarily changed the tendon slack length parameter, while the tuned optimal fiber length remained relatively unchanged. No significant differences (p > 0.05) were found between generic and scaled models for all tested parameters.

**Fig. 6.**
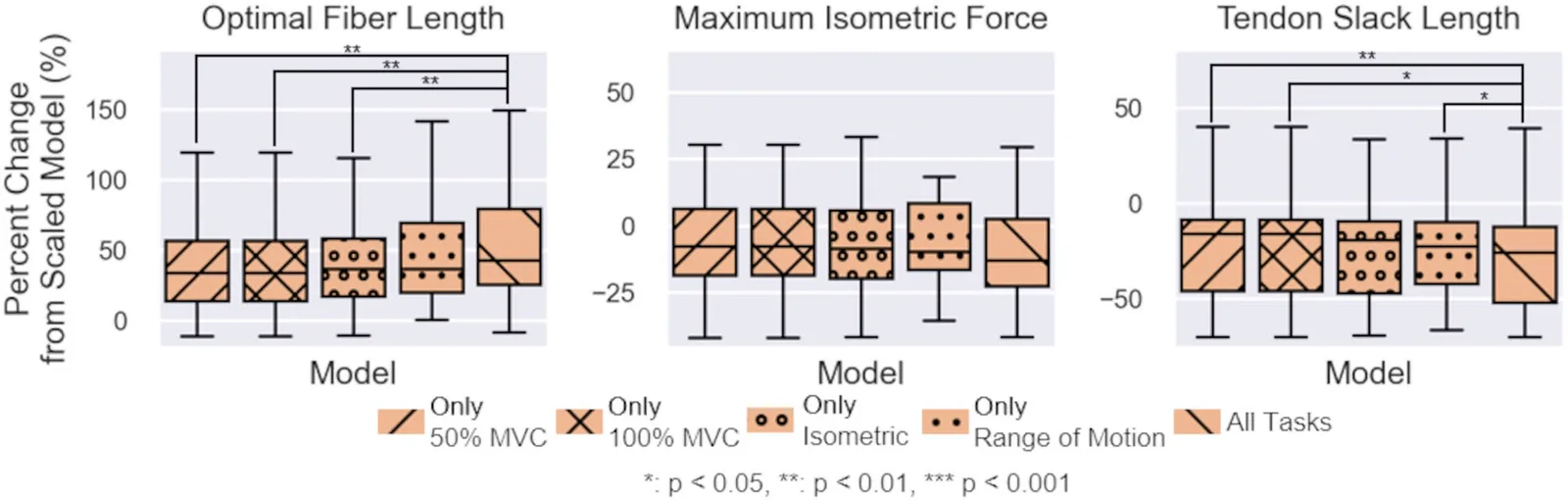
Comparison of training data on NMSM model parameters. The scaled model was chosen as the baseline value since all NMSM models were adapted from the scaled model. The different training sets generally did not lead to significant (p < 0.05) differences in parameters. Training with all tasks was the exception, but initial evaluation of prediction accuracy did not show significant differences from other training sets.

Differences in tuning data did not significantly (p > 0.05) change the NMSM parameter sets (Fig. 6). No significant differences (p > 0.05) were found across tuning data approaches for the personalized maximum isometric force. Furthermore, limited differences were found across tuning data approaches for optimal fiber length and tendon slack length. The tuned optimal fiber length values when using all tasks were significantly higher (p < 0.01) than those derived from tuning with 50% MVC, 100% MVC, or all isometric tasks. Likewise, tuned tendon slack lengths were significantly lower (p < 0.05) when tuning with all tasks compared to only tuning with 50% MVC, 100% MVC, or all isometric tasks.

### B. Prediction Accuracy: Comparison of Muscle Activations

**Fig. 7.**
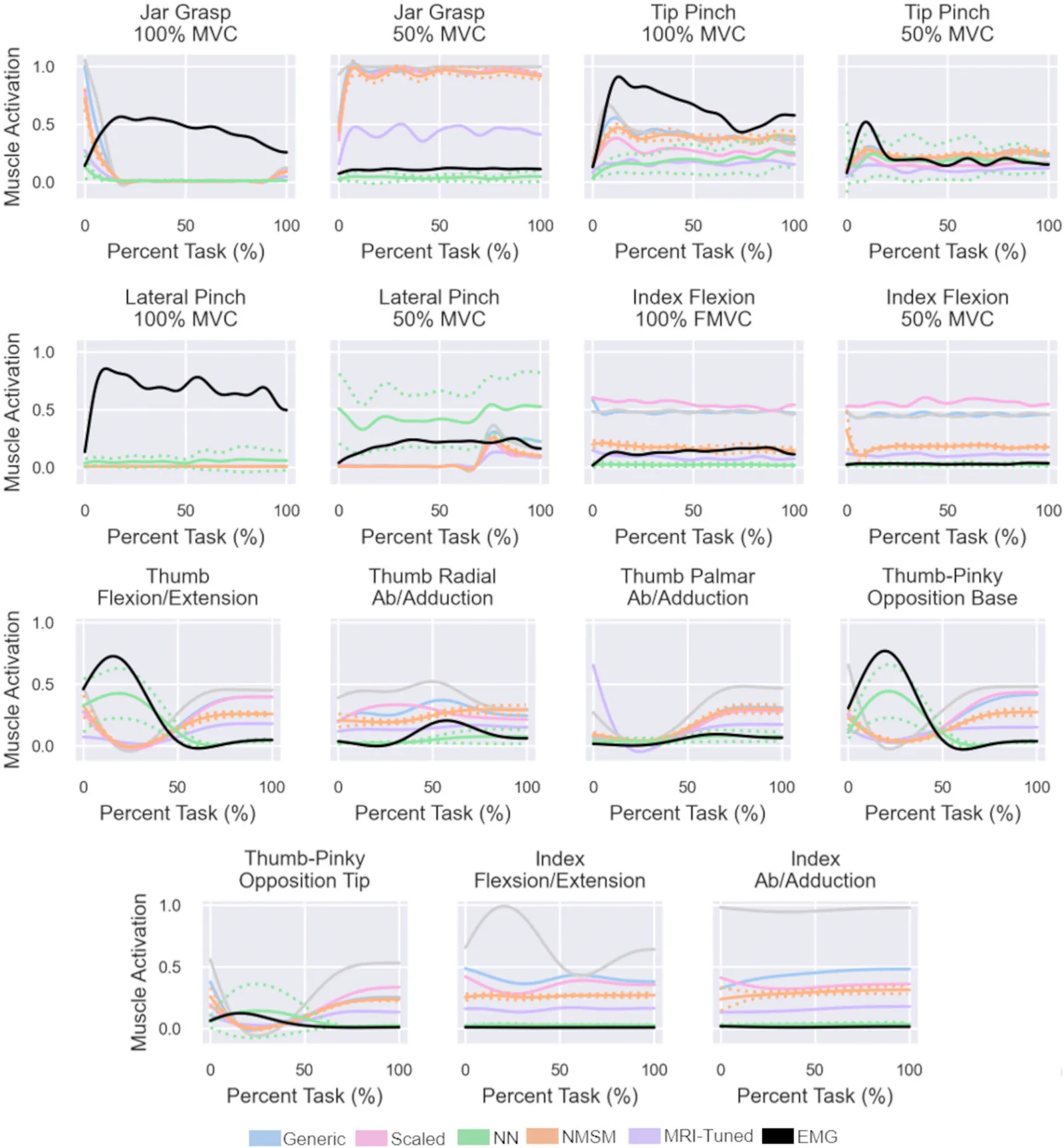
Representative predictions for *flexor pollicis longus* with the measured EMG signal from a single participant and trial. Mean (solid) ±1 standard deviations (dashed) shown for the five predictions performed for NN and NMSM models.

**Fig. 8.**
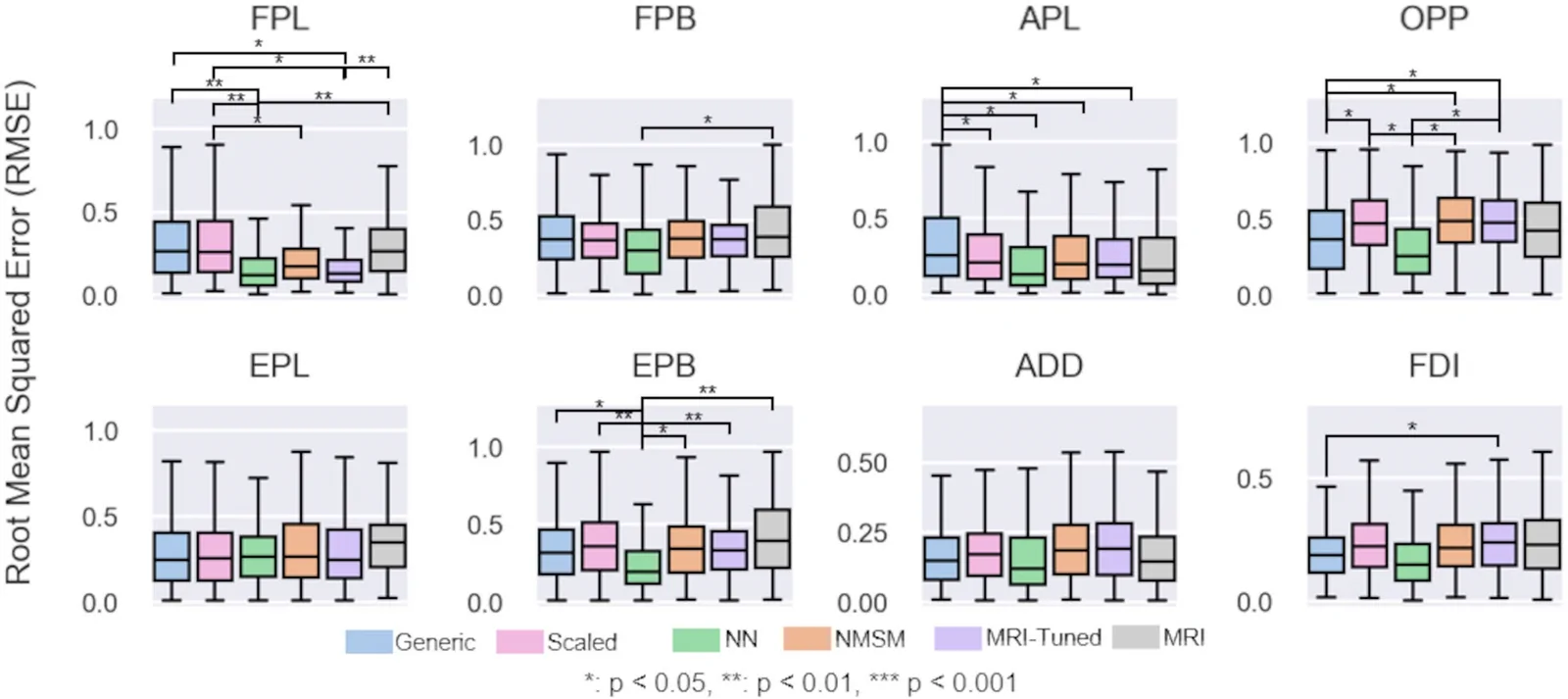
Root mean squared error of predicted muscle activations for different personalized models across all tasks.

Personalizing musculoskeletal models led to significant differences in predicted muscle activations across multiple muscles and tasks when using static optimization. Notably, differences were observed in both shape and magnitude of the muscle activation curves. When looking at shape, there is high variability (Fig. 7) and poor correlations (Supplemental Table 2) between predicted muscle activations and measured EMG. However, it is worth noting that the NN model’s activation curves (Fig. 7) appear to stand apart from the other models, as they often mirror the shape of the other predictions (i.e. when the NN personalized models tend towards increased activations, the other personalization methods tend toward decreased activations). These shape characteristics as well as differences in magnitude are reflected in average RMSE of muscle activations which ranged from 0.16 (FPL: NN, MRI-tuned) to 0.49 (OPP: NMSM) with standard deviations ranging from 0.11 (FDI: NN, Generic) to 0.26 (APL: Generic, MRI) (Table I). Across all models, personalization resulted in significant (p < 0.05) differences for 6 of the 8 evaluated muscles (Fig. 8). The NN models (Fig. 8, green) had significantly lower average RMSE for 5 muscles [APL (mean ±SD: 0.23 ± 0.24), EPB (mean ±SD: 0.24 ±0.27), FPL (mean ±SD: 0.16 ±0.13), FPB (mean ±SD: 0.31 ± 0.21), and OPP (mean ±SD: 0.30 ±0.2)]. Personalizing hand models with NMSM (Fig. 8, orange) also improved predicted muscle activations compared to generic or scaled models for FPL (mean ±SD: 0.21 ±0.16), APL (mean ±SD: 0.27 ± 0.22), and EPB (mean ±SD: 0.37 ±0.22) (p < 0.05). Interestingly, no significant differences (p > 0.05) were seen between the MRI (Fig. 8, grey), generic (Fig. 8, blue), and scaled (Fig. 8, pink) models. Likewise, no significant differences (p > 0.05) existed between the MRI-tuned models (Fig. 8, purple) and those generated by only performing NMSM for model personalization (Fig. 8, orange).

**Fig. 9.**
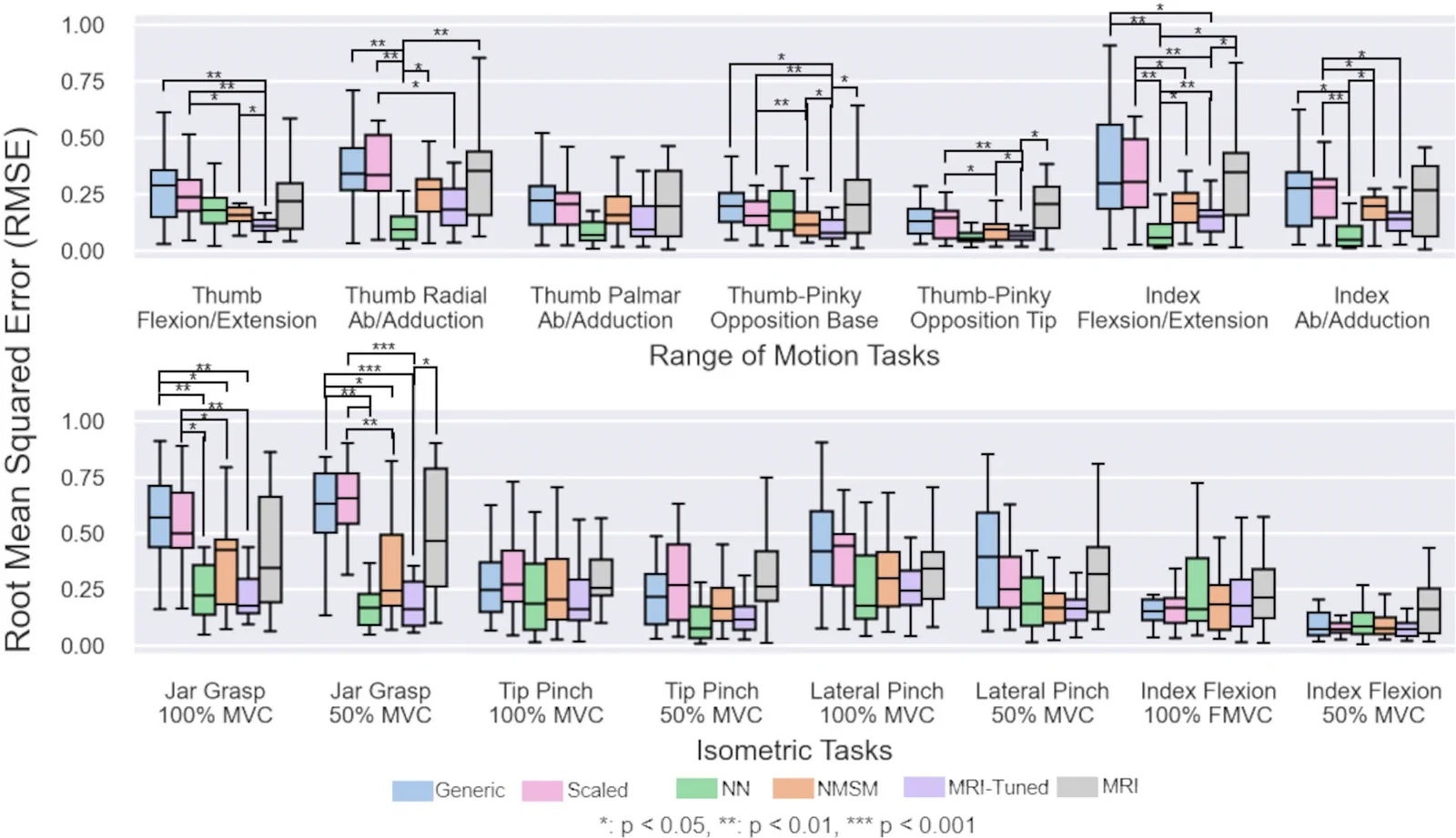
Root mean squared error of predicted *flexor pollicis longus* activations for different personalized models and tasks.

Model personalization had a greater influence on predicted FPL activations during range of motion tasks than during isometric tasks (Fig. 9). Significant differences (p < 0.05) between personalization methods were found for six of seven range of motion tasks. The NN (green), NMSM (orange), and MRI-tuned (purple) methods generally lead to significantly lower (p < 0.05) RMSE compared to using only generic (blue), scaled (pink), or MRI (grey) models (Fig. 9). Furthermore, the NN and MRI-tuned models had the lowest average RMSE (0.07), followed by NMSM models (0.09), respectively for four of the tested range of motion tasks (thumb radial ab/adduction, thumb-index opposition tip, index flexion/extension, index ab/adduction). While significant differences in RMSE were identified for five (Lateral Pinch 100% MVC and 50% MVC, Grip 100% MVC and 50% MVC, and Tip Pinch 50%MVC) of the eight isometric tasks, post hoc testing only showed differences during isometric grip tasks. Specifically, NN models (green) and MRI-tuned models (purple) had significantly lower RMSE in predict FPL activation compared to other personalization methods (Fig. 9).

## IV. Discussion

Anatomically accurate musculoskeletal model parameters are not always necessary for improved model performance. Although the NN approach had the greatest error in parameter values when compared to the parameter derived from MRI (Fig. 5), the NN approach provided comparable RMSE in predicted muscle activations across tasks. Personalized modeling techniques are typically based on adjusting an anatomically derived initial parameter set (e.g., length scaling, MRI derived, or optimizing scaled models), but Hill-type muscle parameter interactions combined with high levels of muscle redundancy in the upper limb provide a semi-infinite solution space. The NN approach leverages the potential for redundant solutions using a non-anatomically accurate parameter set to obtain improved prediction performance. The reverse is also possible where an anatomically accurate parameter set leads to decreased prediction accuracy due to the assumptions within a modeling software (Fig. 7, 8, and 9). Therefore, an advantage of building the NN personalization method from OpenSim simulations is that it can learn how parameters sets, OpenSim’s underlying assumptions, and OpenSim’s default neural control strategies interact to produce predictions. This allows the NN approach to achieve generally more accurate muscle activation predictions without also having to optimize neural control strategies. This is a potential advantage over other methods when it is not feasible to obtain the additional data to optimize both the musculoskeletal and neural control parameters. This is especially important at the hand where the time constraints and invasive fine-wire EMG make it challenging to obtain the data needed to optimize neural controls.

Applying uniform parameter changes across muscles was as effective as applying unique changes to individual muscles. The NN modeling approach had improved performance even though it applied uniform parameter changes to a generic model. This is contrary to previous techniques that apply unique changes to each muscle. It is theorized that the success of the uniform parameter change is due to muscles in the forearm and hand being anatomically connected. This connection is due to muscles crossing multiple joints and the need for coactivation to stabilize the hand during tasks [29], [30], [58]. Thus, unless muscles groups are isolated, hand muscles generally experience similar levels of strain leading to similar rates of hypertrophy or atrophy [59], [60], [61], [62], [63].

The NMSM model tuning pipeline also improved prediction accuracy for upper limb inverse simulations. The joint moment optimization approach which NMSM is built upon has previously been limited to lower limb applications and forwards simulation [14], [15], [20], [45], [64]. However, here we showed that NMSM does significantly improve prediction accuracy for complex interactions during isometric and range of motion tasks at the hand. Furthermore, the EMG-driven forwards tuning approach adapted well to predicting muscle activations, which has a larger solution space. This study also shows that the task data used for NMSM tuning did not significantly (p > 0.05) change muscle parameters (Fig. 6). The exception to this was tuning with data from all 15 tasks, which created significantly (p < 0.05) higher optimal fiber lengths and lower tendon slack lengths compared to tuning with either 100% MVC or 50% MVC tasks. However, the similarity in parameter sets and prediction accuracy demonstrates the possibility to personalize hand musculoskeletal models with limited tasks.

**Fig. 10.**
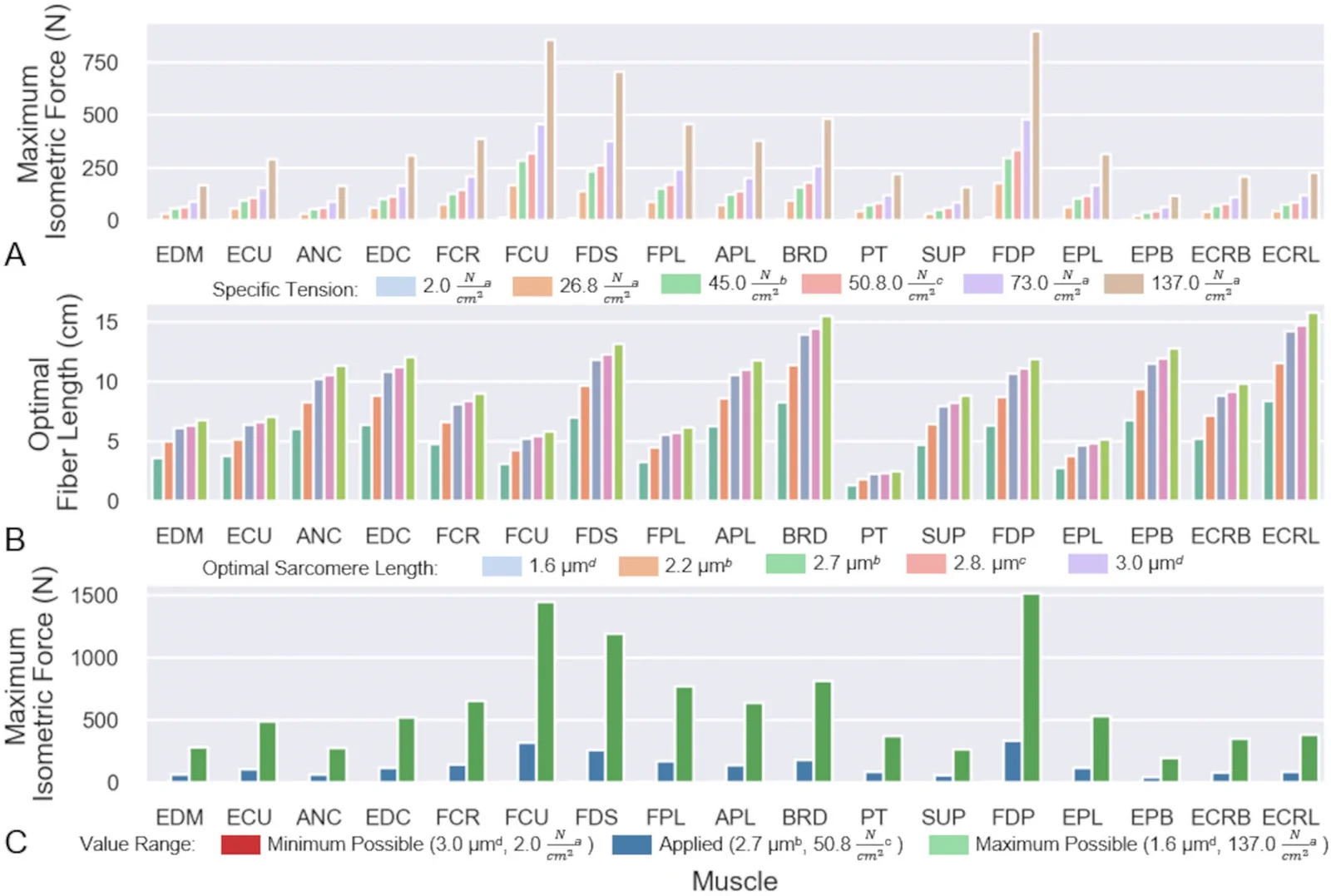
Influence of assumed optimal sarcomere length and specific tension on calculate maximum isometric force and optimal fiber length for 17 muscles from a representative subject. A) Range of possible maximum isometric forces based on reported literature values for specific tension. B) Range of possible optimal fiber lengths based on reported literature values for optimal sarcomere length. C) Extreme values for maximum isometric force by varying assumptions of optimal sarcomere length and specific tension to minimize or maximize maximum isometric force, with the applied assumptions shown in blue.

A lack of robust anatomical gold standard limits the ability to define anatomical accuracy of musculoskeletal parameters. Although many researchers consider MRI derived parameters to be the personalized musculoskeletal modeling ‘gold standard’, the need for assumed optimal sarcomere length and specific tension mean muscle parameters can vary widely (Fig. 10) [26], [65]. Therefore, MRI derived parameters rely heavily on how well key assumptions fit a participant, leading to the possibility of high errors and variability. For example, reported literature values of optimal sarcomere length and specific tension can be maximized to create the largest maximum isometric force, or minimized to create the lowest value for maximum isometric force (Fig. 10). This large range highlights that MRI personalization should not be considered a ‘gold standard’ because of variability from assumed optimal sarcomere length and specific tension. Accuracy of MRI values can also be influenced by the quality of the MRI scans, which can be affected by equipment, participant characteristics, slice thickness, and segmentation software limitations.

The ability of personalization methods to generate different parameter sets emphasizes the need to understand how accurate Hill-type muscle model parameters need to be. Previous work highlights that, within the Hill-type muscle model, parameter values are either non-essential or carry poor assumptions [26], [66]. For example, pennation angle has been shown to not influence musculoskeletal simulations and assumed values of specific tension are known to change between both muscles and people [26], [66]. These shortcomings present the issue of determining whether anatomically accurate Hill-type muscle parameters are necessary for musculoskeletal modeling. The effort required to personalize models could be time ill-spent, if the increased anatomical accuracy leads to decreased prediction accuracy. In a similar manner, a model personalized to increase prediction accuracy at the expense of anatomical accuracy could be ineffective if the intent is to understand anatomical changes. Therefore, a deeper understanding of the relationship between anatomical and prediction accuracy is needed to determine ideal methods for model personalization.

As with all musculoskeletal modeling approaches, this study is affected by some limitations. Despite significant findings, high variance existed across participants. The high variance is expected as i) the muscle redundancy causes the solution space to be semi-infinite for inverse simulations and ii) neural controls were not personalized. Neural controls were purposefully not personalized since neural control tuning often uses similar tasks for training and testing models which can bias results as the model is personalized using data that is nearly identical to the testing data. Therefore, further analysis is needed to understand the hand’s heterogeneous muscle recruitment strategies, how these strategies influence prediction accuracy, and how best to implement neural control models in the hand. While normalized EMG can be used to evaluate predictions of muscle activations [42], [56], it should be acknowledged that normalized EMG is not exactly equal to muscle activations due to the challenges of correctly incorporating electromechanical delay [56] and/or eliciting true MVCs for normalization [42]. These limitations could cause errors in calculated RMSE. While this work did not explore sensitivity to incorporating electromechanical delay and/or various normalization methods, based on the large differences in magnitude and shape in muscle activation curves (Fig. 7), it is unlikely either of these limitations alone account for the observed errors. This work also does not explore how NMSM’s use of a non-compliant tendon model within its internal optimizer influences studies, like this one, that rely on models that use a different muscle model. Instead, this study aimed to test the generalizability of NMSM, the influence of training data on NMSM, and if NMSM could even be applied to standard upper limb models. Due to the constraints of MRI, the sample size was limited to 13 individuals, and further recruitment may have allowed for identifying participant characteristics that lead to improved performance across or within personalization approaches. MRI personalization was also limited to only Hill-type muscle model parameters and did not consider muscle attachments or paths.

Personalization time varied across methods from 0.6 seconds up to 56 hours. The NN method provided the lowest prediction error and allowed for models to be created in 0.6 seconds per participant and required input of only height, weight, and a 5 second lateral pinch measurement. While this does not factor in the time and computational resources required to develop the NN pipeline, the method’s task generalizability shows potential to not need a unique neural network for each task. In contrast, NMSM tuning also provided improved prediction accuracy but required 48 hours of data preprocessing due to the need for additional simulations and data formatting. The MRI personalization method provides a middle ground taking approximately 8 hours of manual segmentation; however, the improved anatomical accuracy did not improve prediction accuracy compared to generic and scaled models, which can be created in less than an hour.

This study compared a novel machine learning method to current state-of-the-art methods for musculoskeletal model personalization. The NN personalization method was comparable to current methods when compared with a measured MRI and fine-wire EMG data. The comparison covered the hand’s diverse functions using 15 unique hands tasks. Future work is needed to understand how personalizing for anatomical versus prediction accuracy influences modeling decisions across both the upper and lower limb.

## Supplementary Material

supp2-3711799

supp1-3711799

This article has supplementary downloadable material available at https://doi.org/10.1109/TNSRE.2026.3711799, provided by the authors.

## Figures and Tables

**TABLE I:** Average Model RMSE Across all Static Optimization Tasks

Model	FPL RMSE Mean (SD)	FPB RMSE Mean (SD)	APL RMSE Mean (SD)	OPP RMSE Mean (SD)	EPL RMSE Mean (SD)	EPB RMSE Mean (SD)	ADD RMSE Mean (SD)	FDI RMSE Mean (SD)

Generic	0.32 (0.23)	0.40 (0.21)	0.34 (0.26)	0.38 (0.23)	0.29 (0.21)	0.34 (0.2)	0.18 (0.13)	0.20 (0.11)
Scaled	0.30 (0.22)	0.42 (0.23)	0.27 (0.26)	0.43 (0.24)	0.34 (0.19)	0.42 (0.24)	0.18 (0.13)	0.24 (0.13)
NN	0.16 (0.12)	0.38 (0.19)	0.26 (0.21)	0.48 (0.19)	0.30 (0.21)	0.35 (0.21)	0.21 (0.14)	0.24 (0.12)
NMSM	0.21 (0.16)	0.39 (0.2)	0.27 (0.22)	0.49 (0.21)	0.31 (0.22)	0.37 (0.22)	0.20 (0.13)	0.23 (0.13)
MRI-Tuned	0.16 (0.13)	0.31 (0.21)	0.23 (0.24)	0.30 (0.2)	0.28 (0.17)	0.24 (0.17)	0.17 (0.14)	0.17 (0.11)
MRI	0.30 (0.21)	0.38 (0.19)	0.28 (0.23)	0.48 (0.21)	0.29 (0.2)	0.38 (0.23)	0.19 (0.13)	0.23 (0.12)
