# Supplementary material for: Benchmarking Neural Network Personalized Musculoskeletal Hand Models Against Current Personalization Standards Using Experimental Magnetic Resonance Imaging and Fine-Wire Electromyography
Source: IEEE Trans Neural Syst Rehabil Eng. Author manuscript; Available in PMC 2026 Sep 8. (PMC13551466; doi:10.1109/TNSRE.2026.3711799)
Supplement: Supplementary file 2 [file NIHMS2200135-supplement-supp2-3711799.docx]

**Supplemental Table 2. Pearson’s correlation coefficient squared (r^2^) for Muscle Activation Curves^1^**

| Task | Generic | Scaled | NN | NMSM | MRI-Tuned | MRI |
| --- | --- | --- | --- | --- | --- | --- |
| Thumb  Flexion/Extension | 0.19 (0.2) | 0.18 (0.2) | 0.39 (0.27) | 0.27 (0.24) | 0.26 (0.25) | 0.21 (0.22) |
| Thumb  Radial Ab/Adduction | 0.33 (0.24) | 0.29 (0.24) | 0.25 (0.22) | 0.28 (0.21) | 0.29 (0.22) | 0.26 (0.26) |
| Thumb Palmar Ab/Adduction | 0.22 (0.2) | 0.27 (0.23) | 0.21 (0.2) | 0.21 (0.2) | 0.24 (0.2) | 0.21 (0.18) |
| Thumb-Pinky  Opposition Base | 0.23 (0.22) | 0.24 (0.2) | 0.43 (0.29) | 0.23 (0.24) | 0.27 (0.25) | 0.3 (0.22) |
| Thumb-Pinky  Opposition Tip | 0.23 (0.18) | 0.27 (0.21) | 0.24 (0.2) | 0.25 (0.24) | 0.3 (0.23) | 0.25 (0.21) |
| Index  Flexion/Extension | 0.21 (0.19) | 0.16 (0.17) | 0.18 (0.19) | 0.24 (0.16) | 0.23 (0.19) | 0.16 (0.18) |
| Index  Ab/Adduction | 0.18 (0.2) | 0.14 (0.13) | 0.22 (0.19) | 0.18 (0.2) | 0.11 (0.13) | 0.17 (0.18) |
| Jar Grasp  100% MVC | 0.19 (0.16) | 0.16 (0.15) | 0.17 (0.2) | 0.25 (0.24) | 0.19 (0.21) | 0.12 (0.11) |
| Jar Grasp  50% MVC | 0.21 (0.21) | 0.22 (0.21) | 0.16 (0.21) | 0.24 (0.21) | 0.11 (0.12) | 0.13 (0.15) |
| Tip Pinch  100% MVC | 0.28 (0.22) | 0.28 (0.21) | 0.2 (0.21) | 0.29 (0.22) | 0.22 (0.18) | 0.2 (0.18) |
| Tip Pinch  50% MVC | 0.26 (0.23) | 0.31 (0.23) | 0.19 (0.23) | 0.33 (0.25) | 0.25 (0.24) | 0.24 (0.23) |
| Lateral Pinch  100% MVC | 0.18 (0.17) | 0.21 (0.19) | 0.2 (0.2) | 0.22 (0.2) | 0.2 (0.2) | 0.21 (0.18) |
| Lateral Pinch  50% MVC | 0.21 (0.22) | 0.22 (0.21) | 0.18 (0.19) | 0.24 (0.21) | 0.21 (0.17) | 0.19 (0.18) |
| Index Flexion  100% MVC | 0.11 (0.13) | 0.12 (0.16) | 0.15 (0.16) | 0.14 (0.15) | 0.11 (0.14) | 0.12 (0.14) |
| Index Flexion  50% MVC | 0.14 (0.15) | 0.13 (0.13) | 0.17 (0.18) | 0.14 (0.15) | 0.1 (0.12) | 0.11 (0.13) |

^1^Reported as Mean (Standard Deviation)
