# Supplementary material for: Benchmarking Neural Network Personalized Musculoskeletal Hand Models Against Current Personalization Standards Using Experimental Magnetic Resonance Imaging and Fine-Wire Electromyography
Source: IEEE Trans Neural Syst Rehabil Eng. Author manuscript; Available in PMC 2026 Sep 8. (PMC13551466; doi:10.1109/TNSRE.2026.3711799)
Supplement: Supplementary file 3 [file NIHMS2200135-supplement-supp1-3711799.docx]

**Supplemental Table 1. Opensim model muscles calculated from MRI segmentations**
